# Insecticide-treated bed net utilization and its determinants among pregnant women in Dembecha District, Northwest Ethiopia

**DOI:** 10.1186/s41182-025-00762-0

**Published:** 2025-10-01

**Authors:** Abraham Teym, Gete Berihun, Mestet Yibeltal Shiferaw, Etsubdink Dessalew Abawa, Yibeltal Alemu, Bayou Tilahun Assaye, Rahel Belete Abebe, Tirsit Ketsela Zeleke

**Affiliations:** 1 Department of Environmental Health, College of Health Sciences, Debre Markos University, Debre Markos, Ethiopia; 2 Department of Surgery, Neurosurgery Unit, Injibara University, Injibara, Ethiopia; 3 Department of Nursing, College of Medicine and Health Sciences, Debre Markos University, Debre Markos, Ethiopia; 4 Department of Medical Laboratory Sciences, College of Medicine and Health Sciences, Debre Markos University, Debre Markos, Ethiopia; 5 Department of Health Informatics, College of Medicine and Health Sciences, Debre Markos University, Debre Markos, Ethiopia; 6 Department of Pharmacy, College of Medicine and Health Sciences, University of Gondar, Gondar, Ethiopia; 7 Department of Pharmacy, College of Health Sciences, Debre Markos University, Debre Markos, Ethiopia

**Keywords:** Insecticide-treated bed nets, Utilization, Malaria prevention, Vector control, Antenatal care, Pregnant women, Ethiopia

## Abstract

**Background:**

Insecticide-treated nets (ITNs) are widely used and proven effective in preventing and controlling malaria. However, their utilization varies among households, which can significantly impact the benefits of insecticide-treated nets. This study aimed to assess the household utilization of ITNs and the associated factors among pregnant women.

**Methods:**

A community-based cross-sectional study was conducted from April to May 2024, including 415 randomly selected pregnant women. Data collection employed a pretested questionnaire, and logistic regression analysis was utilized to identify factors influencing insecticide-treated net (ITN) usage. Variables with a *p*-value < 0.25 in the univariable logistic regression were considered as candidate variables for inclusion in the multivariable logistic regression model. Adjusted odds ratios (AORs) with 95% confidence intervals (CIs) were computed, and statistical significance was set at *p* ≤ 0.05. The model’s performance was assessed using the Hosmer–Lemeshow goodness-of-fit test.

**Results:**

The utilization of insecticide-treated bed nets among pregnant women was 46.5% (95% CI: 41.7–51.3%). Pregnant women without formal education (AOR = 0.48; 95% CI: 0.28–0.81), monthly income (AOR = 0.98; 95% CI: 0.44–1.97), pregnant women with a family size of less than five (AOR = 2.53; 95% CI: 1.61–3.87), and pregnant women who attended at least one antenatal care (ANC) visit (AOR = 2.08; 95% CI: 1.21–2.58) were significantly associated with insecticide-treated bed net utilization.

**Conclusion:**

Utilization of insecticide-treated bed nets by pregnant women was 46.5%, which was lower than the WHO recommendation (80%). Insecticide-treated bed nets utilization was significantly associated with education, monthly income, antenatal care (ANC) attendance, and family size. Targeted interventions in Dembecha District should include community training by health extension workers, strengthened household-level bed net supervision, and culturally tailored awareness campaigns via local media and health professionals.

## Introduction

Malaria remains one of the most significant global health challenges, disproportionately affecting tropical and sub-tropical regions, with sub-Saharan Africa bearing the highest burden [1–3]. It is a parasitic disease caused by Plasmodium species, transmitted through the bites of infected female Anopheles mosquitoes [4]. Globally, malaria continues to pose severe health, social, and economic challenges. It is particularly devastating in sub-Saharan Africa, where the disease contributes to approximately 25% of all maternal deaths in endemic areas [5, 6]. The effects of malaria extend beyond health, significantly impacting economies by reducing workforce productivity, increasing healthcare costs, and perpetuating cycles of poverty in affected communities [7, 8].

Pregnant women are among the most vulnerable groups affected by malaria, as pregnancy suppresses immune function, making them more susceptible to infections [7, 9]. This vulnerability is most pronounced in women during their first pregnancies (primigravidae) and those of younger maternal age, who exhibit less acquired immunity than older, multiparous women [10]. Studies estimate that approximately 25 million pregnancies annually are at risk of malaria infection in sub-Saharan Africa alone, with dire consequences for both maternal and fatal health [11, 12].

Malaria during pregnancy is associated with a range of adverse outcomes, including maternal anemia, placental malaria, intrauterine growth restriction (IUGR), and increased risks of miscarriage, stillbirth, and neonatal mortality [13, 14]. Placental malaria, caused by the sequestration of *P. falciparum*-infected erythrocytes in the placenta, leads to placental insufficiency. This compromises the transfer of oxygen and nutrients to the fetus, often resulting in low birth weight (LBW) deliveries, which are a leading cause of neonatal death and long-term developmental issues [14]. Infants born with LBW are at higher risk of delayed social and cognitive development, neonatal sepsis, and mortality in the first year of life [15].

Although ITNs are highly effective, their use by pregnant women in sub-Saharan Africa including Ethiopia, where 65% of the population lives in endemic areas and 75% of the land supports transmission remains alarmingly low [16]. These alarming statistics underscore the urgent need for effective preventive strategies to protect pregnant women and their unborn children from this devastating disease. One of the most effective strategies for malaria prevention is the use of insecticide-treated bed nets (ITNs). Recognizing the critical importance of ITNs, the World Health Organization (WHO) recommends that all pregnant women sleep under an ITN as early as possible during pregnancy, ideally before conception [16, 17].

In endemic areas, consistent and proper use of insecticide-treated nets (ITNs) can reduce malaria episodes by 48–50% and decrease malaria-related deaths by about 20%, as they provide both a physical barrier and insecticidal effect against mosquitoes [18]. For pregnant women, ITN use is particularly crucial as it significantly reduces the risk of adverse pregnancy outcomes. Studies indicate that ITN usage can reduce malaria transmission by up to 90%, miscarriages by 33%, and stillbirths by 25% [16, 19–21].

Studies have identified several barriers to ITN usage, including limited access to ITNs, lack of awareness about their benefits, and sociocultural factors [22]. Educational status, occupation, rural residence, ownership of a television or radio (a proxy for health information exposure), religion, ethnicity, and family income are significant determinants of ITN utilization among pregnant women [23, 24]. Furthermore, misconceptions about ITN safety during pregnancy and discomfort associated with sleeping under nets contribute to low usage rates [16, 25].

In Ethiopia, the Federal Ministry of Health has made substantial efforts to improve ITN access, distributing large quantities of insecticidal treated bed nets (ITN) to address the population at risk [26]. However, this distribution effort has not fully translated into widespread ITN usage, particularly among vulnerable populations such as pregnant women. Research shows that knowledge gaps regarding ITN use, coupled with logistical challenges in distribution, have hindered the success of malaria prevention programs in rural and underserved areas. This study addresses these critical gaps by specifically targeting pregnant women in a rural district through a community-based cross-sectional design. It incorporates a comprehensive set of variables including knowledge, attitudes, access, household characteristics, and decision-making autonomy to provide a nuanced understanding of the determinants of ITN utilization. As such, it offers meaningful, context-specific evidence to inform more targeted and effective malaria prevention strategies in similar rural settings [27–29]. Therefore, this study aimed to assess the utilization and associated factors of insecticide-treated nets among pregnant women in Dembecha District, which helps guide policymakers and concerned bodies to emphasize the utilization of ITNs by pregnant women.

## Methods and materials

### Study area and period

The study was conducted in Dembecha district, in the Amhara Region, from April 2024 to May 2024. Dembecha is a district in north-western Ethiopia, 350 km north of Addis Ababa. Located in the West Gojjam Zone of the Amhara Region, this town has a latitude and longitude of 10°33′N and 37°29′E with an elevation of 2083 m above sea level. The district has 19 health centers and 4 private clinics [30]. Dembecha District comprises 33 kebeles. For this study, data were collected from ten selected kebeles: Yezeleka, Kendamo, Egziarab, Adiszemen, Jajirab, Mekar, Asteboj, Gedeb, Wejet, and Enwond. The selection was based on malaria risk levels and accessibility to health services, which are key factors influencing the use of insecticide-treated nets (ITNs). According to the 2023 District Health Office report, malaria contributed to approximately 16% of antenatal care-related outpatient visits, with increased incidence during the rainy season. This underlines the importance of promoting effective malaria prevention strategies, including insecticide-treated bed net utilization, among this high-risk group.

### Study design and population

This community-based cross-sectional study was conducted among pregnant women in Dembecha District. Participants were identified and recruited using registration records maintained by health extension workers at each kebele health post, which are regularly updated through house-to-house visits and antenatal care (ANC) follow-ups. The source population included all pregnant women residing in Dembecha District, while the study population comprised pregnant women living in selected kebeles who owned an ITN and were at least six months into their pregnancy. Severely or critically ill pregnant women during the data collection period were excluded from the study.

## Sample size and sampling techniques

### Sample size

The sample size was calculated using a single population proportion formula assuming a 95% confidence interval and 57% prevalence (P) from the study conducted on insecticide treated net utilization among pregnant women in sodo zuria woreda Southern Ethiopia, 2022, and a precision of 5% between the sample and the 10% non-response rate was taken [31]:

$$ n_{i} = \frac{{(Z\alpha /2)^{2} (p) \times (1p)}}{{d^{2} }}, $$

where *n*_*i*_ was the initial sample size, *Z*_α/2_ = was the standard

*d*^2^ score value for a 95 % confidence level for two sides of normal distribution = 1.96, *p* = was the prevalence = 0.51, *d* = was margin of error = 0.05:

$$ {\text{nf}} = \frac{{p(1p)(Z\alpha /2)^{2} }}{{0.05^{2} }} = {\text{nf}} = \frac{{0.57(1 - 0.57)(1.96)^{2} }}{{0.05^{2} }} = {\text{nf}} = 377. $$

The study calculated a final sample size of 415, including a 10% non-response rate.

### Sampling procedures

**Fig. 1:**
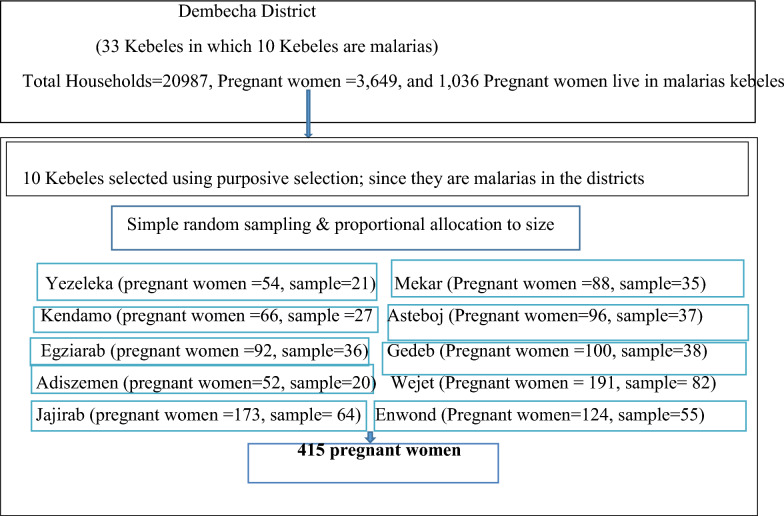
Schematic presentation of sampling procedures

The sample size for each pregnant mother was allocated proportionally to each selected kebeles and each participant was selected using simple random sampling from the sampling frame taking community health information registers as sampling frame (Fig. 1).

### Study variables

The study analyzed ITN utilization as a dependent variable, while independent variables included socio-demographic characteristics, behavioral factors, obstetric factors, and ITN-related factors such as availability, accessibility, affordability, possession of ITNs, and sleeping patterns.

### Operational definition

**Knowledge about malaria and IT:** Respondents were considered as having adequate knowledge (knowledgeable) about malaria if they respond correctly to half of the questions regarding the transmission and prevention methods including the use of ITN and good knowledge (i.e., a score ≥mean score) and poor knowledge (i.e., a score of <the median score [32].

**Attitude towards malaria and ITN:** Assessment of the predisposition to respond favorably or unfavorably towards malaria. 8 items of 5 5-point Likert scale (ranges from strongly agree to strongly disagree) will be used to evaluate the respondents’ attitude towards malaria preventive measures including ITNs. The respondents will be evaluated for the whole items to say low, moderate, and high attitude towards malaria and if the individual gets less than the whole item mean score classified as low attitude, if it is between 50 and 80% considered as moderate attitude and above 80% as high attitude towards malaria.

**ITN utilization:** ITN utilization was assessed through self-reports from currently pregnant women and verified using an observation checklist. A woman was considered to have utilized an ITN if she reported sleeping under it the night before the survey and if the ITN was observed to be hanging and properly tucked around the sleeping area. This measurement followed the standard definition provided by the WHO and the Roll Back Malaria Monitoring and Evaluation Reference Group (MERG), which defines ITN use as sleeping under a net the previous night [33].

### Data collection tool

Following a study of the relevant literature, an interviewer-administered questionnaire was developed for gathering data. It was originally written in English and then translated into the local language, Amharic, for data collecting before being translated back into English for consistency. The questionnaire, which is administered in Amharic, asked about socio-demographic data, knowledge, health information, and other associated areas. Data collectors are selected based on their ability to understand the local languages, their willingness to participate in the survey, and their manners. Six data collectors and two supervisors got one-day training on how to collect data prior to the actual data collection sessions. The training focuses on the survey’s aims, the significance of each question, interview techniques, data confidentiality, informed consent, and the roles and responsibilities of data collectors and supervisors. Data were obtained using a specific format, which included several factors. The questionnaire was filled out by trained data collectors.

### Data quality management

To ensure valid information, the questionnaire was first developed in English, then translated to the local language Amharic, and then back to English. Before actual data collection, questionnaires were pretested in Dembecha, district, on those Kebeles which was not included in sampling area. The pretest was done on 5% of the total sample size to ensure the clarity, ordering, consistency and acceptability of the questionnaire. One day training of data collectors and supervisors was conducted to ensure the quality of data. The collected data were reviewed by data collectors and supervisors for completeness and logical consistency. Additionally, the collected data also checked by the principal investigator every day. Finally, data cleaning was conducted at the end of data entry.

### Data processing and analysis

The collected data were entered, cleaned, checked and coded using Epi data version 3.1, and then exported to SSPS version 25 for analysis. Descriptive statistics was used to describe the variables by using presented by text, tables, and graph. binary logistic regression was first used to estimate crude odds ratios (COR) with 95% confidence intervals for each independent variable; those with *p* < 0.25 in bivariable analysis were entered into a multivariable model to calculate adjusted odds ratios (AOR). We have defined the reference category for each categorical predictor, assessed multicollinearity using variance inflation factors, and evaluated model fit with the Hosmer–Lemeshow test (*p* = 0.56). Statistical significance is now explicitly reported at *p* < 0.05 for all AORs.

## Results

### Socio-demographic characteristics of study participants

In this study, a total of 413 pregnant women participated, resulting in a response rate of 99.5%. Among the participants, 90.3% were from rural communities, and 97.7% were married. More than half (68%) had attended primary education, while 73.1% worked as housewives. Approximately 60% of the respondents had five or more family members. Detailed socio-demographic characteristics of the participants are shown in Table 1.

**Table 1:** Socio-demographic characteristics of study participants among pregnant women in Dembecha District of West Gojjam Zone, 2024 (*N* = 413)

Variables	Category	Frequency (%)
Residence	Semi-urban	40 (9.7)
	Rural	373 (90.3)
Religion	Orthodox	14 (3.4)
	Muslim	380 (92.0)
	Protestant	19 (4.6)
Marital status	Married	402 (97.3)
	Divorced	11 (2.7)
Pregnant women education	No formal education	101 (24.5)
	Primary (1–8)	284 (68.8)
	Secondary (9–12)	21 (5.1)
	College and above	7 (1.7)
Pregnant women occupation	House wife	302 (73.1)
	Merchant	87 (21.1)
	Day laborer	14 (3.4)
	Government employed	10 (2.4)
Family size	Less five member	165 (40)
	Five and above	248 (60)
Monthly income (ETB)	<500	86 (20.8)
	500–1000	131 (31.7)
	≥1000	196(47.5)

### Maternal characteristics

Among the 413 pregnant women included in the study, 44.8% were primipara, while 55.2% were multipara. Regarding gravidity, 56.2% were primigravida, and 43.8% were multigravida. A majority of the participants (80.1%) had attended at least one antenatal care (ANC) visit, with the majority of these visits occurring during the second trimester (56.2%) or third trimester (28.8%) at the time of data collection (Table 2).

**Table 2:** Maternal characteristics of study participants among pregnant women in Dembecha District of West Gojjam Zone, 2024 (*N* = 413)

Variables	Category	Frequency (%)
Parity	Primipara	185 (44.8)
	Multipara	228 (55.2
Gravidity	Prim-gravida	232 (56.2)
	Multigravida	181 (43.8)
Stage of current pregnancy	1st trimester	62 (15.0)
	2nd trimester	232 (56.2)
	3rd trimester	119 (28.8)
History of abortion	Yes	8 (1.9)
	No	405 (98.1
ANC attended	Yes	331 (80.1)
	No	82 (19.9)
ANC visit rounds (*N* = 331)	1st visit	195 (58.9)
	2nd visit	106 (32.0)
	3rd visit or more	30 (9.1)

### Attitude towards ITN utilization

The study assessed participants’ attitudes toward ITN utilization using eight questions, as detailed in the following table. Of the 413 participants, 65.86% of pregnant women had a positive attitude toward ITN utilization, while 34.14% had a negative attitude (Table 3).

**Table 3:** The study participant’s attitude towards ITN utilization among pregnant women in Dembecha District of West Gojjam Zone, 2024 (*N* = 413)

Statements	Level of agreement	Frequency (%)	SD, mean
Malaria is the most serious health problem in the community	Strongly disagree	81 (19.6)	(1.52, 3.49)
	Disagree	42 (10.2)	
	Neutral	28 (6.8)	
	Agree	119 (28.8)	
	Strongly Agree	143 (34.6)	
Malaria can cause a serious health problem for the fetus in the womb	Strongly disagree	70 (16.9)	(1.44, 3.61)
	Disagree	30 (7.3)	
	Neutral	31 (7.5)	
	Agree	142 (34.4)	
	Strongly Agree	140 (33.9)	
Malaria can lead to death if not treated	Strongly disagree	71(17.2)	(1.35, 3.46)
	Disagree	20 (4.8)	
	Neutral	60 (14.5)	
	Agree	171 (41.4)	
	Strongly Agree	91 (22.0)	
Malaria can be transmitted without mosquito bite	Strongly disagree	31 (7.5)	(1.26, 3.69)
	Disagree	51 (12.3)	
	Neutral	76 (18.4)	
	Agree	112 (27.1)	
	Strongly Agree	143 (34.6)	
Stagnant water and marsh areas can facilitate mosquito breeding	Strongly disagree	51 (12.3)	(1.43, 3.68)
	Disagree	60 (14.5)	
	Neutral	20 (4.8)	
	Agree	121 (29.3)	
	Strongly Agree	161 (39.0)	
It is possible to prevent malaria	Strongly disagree	41 (9.9)	(1.33, 3.73)
	Disagree	50 (12.1)	
	Neutral	40 (9.7)	
	Agree	132 (32.0)	
	Strongly Agree	150 (36.3)	
Utilization of ITNs is one of the best methods of malaria prevention	Strongly disagree	51 (12.3)	(1.45, 3.58)
	Disagree	70 (16.9)	
	Neutral	40 (9.7)	
	Agree	91 (22)	
	Strongly Agree	161(39)	
I am interested to use ITNs regularly and to prevent malaria	Strongly disagree	51 (12.3)	(1.26, 3.69)
	Disagree	60 (14.5)	
	Neutral	60 (14.5)	
	Agree	132 (31.0)	
	Strongly Agree	110 (26.0)	
Attitude	Positive	272 (65.9)	(1.21, 3.58)
	Negative	141 (34.1)	

### Knowledge of pregnant women on malaria and ITN

Most respondents (90.3%) recognized malaria as a communicable disease, and 83.9% correctly identified mosquito bites as the primary transmission mode. While 90.5% had heard of ITNs, only 26.8% identified them as an effective preventive measure. Nearly half (44.6%) of respondents reported having malaria in the past 6 months, with 77.7% seeking treatment at health facilities. Based on correct responses to the key knowledge items, 230 participants (55.7%) were categorized as having good knowledge, while 183 (44.3%) had poor knowledge. This classification was based on a composite score, where respondents answering at least 50% of the knowledge-related questions correctly were considered to have good knowledge (Table 4).

**Table 4:** Knowledge of the study participants on malaria and ITN utilization among pregnant women in Dembecha District of West Gojjam Zone, 2024 (*N* = 413)

Variables	Category	Frequency (%)
Malaria transmitted from person to person	Yes	373 (90.3)
	No	40 (9.7)
Way malaria transmission	Mosquito	313 (83.9)
	Air born	10 (2.7)
	Contact with infected person	40 (10.7)
	Other	10 (2.7)
Knowledge of high-risk group	Yes	205 (49.6)
	No	208 (50.4)
Malaria preventable	Yes	261 (63.2)
	No	152 (36.8)
Methods malaria prevention	IRS	140 (53.6)
	ITN	70 (26.8)
	Herb spray	21 (5.1)
	Taking antimalarial medication	30 (7.3)
Knowledge of benefit of ITN	Yes	155 (37.5)
	No	258 (62.5)
Heard about ITN	Yes	373 (90.3)
	No	40 (9.7)
Source of information about ITN	Radio	10 (2.7)
	Partner or friends	71 (19.0)
	Health workers	282 (75.6)
	School	10 (2.7)
Infected by malaria in the last six months	Yes	184 (44.6)
	No	229 (55.4)
Health seeking during illness (*N* = 165)	Home with modern medicine	28 (15.2)
	Home with traditional	13 (7.1)
	Health facility	143 (77.7)

### Sleeping patterns and ITN utilization

The majority (93.9%) of respondents reported owning an ITN, with 90.5% receiving it for free from health facilities. About 70.9% of households owned only one ITN, and field observations indicated that 33% of the ITNs were in good condition. Regarding ITN utilization, 67.8% were observed hanging over beds during home visits, while 62.6% of pregnant women reported sleeping under an ITN the night before the survey. However, the overall reported ITN utilization in the study setting was only 46.5% (Table 5).

**Table 5:** Sleeping patterns and ITN utilization among pregnant women in the Dembecha District of West Gojjam Zone, 2024 (*N* = 413)

Variables	Category	Frequency (%)
Own ITN	Yes	388 (93.9)
	No	25 (6.1)
Source of ITN [*N* = 388]	Received from health facility	351 (90.5)
	Bought from market	37 (9.5)
Reason for not having	Unable to buy	10 (40)
	Waiting for free supply	10 (40)
	Damaged and old	5 (20)
Number of ITN households owned	One	275 (70.9)
	Two or more	113 (29.1)
Condition of ITN (observed)	Good (no holes)	128 (33)
	Poor (1–4 holes)	260 (67)
Hanged over bed (observed)	Yes	263 (67.8)
	No	125 (32.2)
Utilized ITNs as reported by respondents	Yes	192 (46.5)
	No	221 (53.5)

### Factors associated with ITN utilization among pregnant women

Binary logistic regression was conducted to identify factors associated with ITN utilization, incorporating socio-demographic characteristics, maternal factors, knowledge, attitudes, and ITN-related variables. In the crude analysis, maternal education, parity, ANC attendance, monthly income, and family size were associated with ITN utilization at a *p*-value < 0.25 and were included in the multivariable model.

After adjusting for potential confounders, several factors were significantly associated with ITN utilization among pregnant women. Women with no formal education had lower odds of using ITNs compared to those with at least primary education (AOR: 0.48; 95% CI: 0.28–0.81). Pregnant women who attended antenatal care (ANC) visits were more likely to use ITNs (AOR: 2.08; 95% CI: 1.21–3.58). Family size was also associated with ITN use; women from households with fewer than five members had higher odds of utilization (AOR: 2.53; 95% CI: 1.65–3.87). Monthly income showed a statistically significant association with ITN utilization (AOR: 0.89; 95% CI: 0.44–1.97; *p* < 0.05) (Table 6).

**Table 6:** Multivariable analysis of factors associated with ITN utilization among pregnant women in Dembecha District of West Gojjam Zone, 2024 (*N* = 413)

Variables				COR			AOR		
		Yes	No	COR	95% CI	*P* value	AOR	95% CI	*P*-value
Marital status	Married	190	212	4.033	0.86–18.90	0.077	1.64	0.174–15.47	0.66
	Divorced	2	9	1			1		
Educational status	No formal education	30	71	0.391	0.242–0.633	<0.001	0.479	0.28–0.81	0.002*
	At least Primary	162	150	1			1		
Parity	Primipara	105	80	2.127		<0.06	1.98	1.30–3.03	0.08
	Multipara	87	141	1			1		
ANC attendance	Yes	166	165	2.167	1.298–3.618	0.003	2.08	1.21–3.58	0.001*
	No	98	67	1			1		
Family size	Less than five members	94	154	2.396	1.602–3.585	<0.001	2.53	1.65–3.87	<0.001*
	Five and above	119	154	1			1		
Monthly income (ETB)	<1000	98	119	0.894	0.435–1.968	<0.001	0.9470	1.561–2.153	<0.001*
	≥1000	94	102	1			1		

Keys: * significant at *p*-value less than 0.01

## Discussion

This community-based study assessed insecticide-treated net (ITN) utilization and associated factors among pregnant women in Dembecha District, Northwest Ethiopia. The key finding revealed that 46.5% (95% CI: 41.7–51.3%) of pregnant women reported using an ITN the night before the survey. The study identified educational status**,** monthly income, family size, and antenatal care (ANC) attendance as significant predictors of ITN utilization. This finding is consistent with results from Miesso District Eastern [34], in southern Ethiopia (48%) [28], Ghana (43.3%) [35], Namibia (47%) [36], and with the study conducted in East African Countries 47.05% [37]. This utilization rate is higher than that of other malaria-endemic countries, such as Ethiopia (39.9%) [34] and Uganda (35%) [38]. Higher utilization compared to other reports could be linked to more effective health education interventions or better access to antenatal care services in the study area. However, ITN utilization in this study was lower compared to findings from other regions, such as Sodo Zuria Woreda, Southern Ethiopia (56.89%) [31], Mesekan District, Gurage Zone (58.2%) [39], Dawo District, Southwest Shoa Zone, Oromia (55.5%) [40], Asgede Tsimbla District, Northern Ethiopia (63.1%) [41], Rwanda (75%) [27], and Nigeria (71.8%) [42]. Lower ITN utilization observed in this study compared to others may be attributed to differences in community awareness, distribution strategies, seasonal timing of data collection, or study design. Unlike many previous studies that were institution-based, the current study was conducted at the community level, which may have influenced the findings.

In this study, educational status, monthly income, family size and pregnant women who attended ANC were statistically significant predictors of pregnant women ITN utilization.

Pregnant women with formal education tend to have greater awareness of malaria prevention strategies and are more likely to use ITNs consistently; in this study, those with no formal education were 52% (AOR = 0.48; 95% CI: 0.28–0.81) less likely to utilize ITNs compared to women with at least a primary education. This finding is consistent with studies conducted in Damot Pulasa District, Southern Ethiopia [43], in Shashogo District, Southern Ethiopia [28], and Addis Zemen Hospital, Northwestern Ethiopia [16], which similarly reported higher ITN utilization among educated women. The likely explanation is that educated women possess better knowledge regarding the benefits and correct use of ITNs, and are often more empowered and financially secure, thereby facing fewer barriers such as transportation costs, cultural constraints, and male dominance [37].

Getting greater than 1000 Ethiopian Birr per month was positively associated with ITN utilization in this study. It is consistent with study from south west Ethiopia [44] and Ilu Galan District, Oromia Region, Ethiopia [45] where households with higher wealth indexes were more likely to utilize ITN. This is because those households that have better income can afford ITNs and utilize than those with low monthly income. This result is inconsistent with a study conducted in Ghana [46]. This may be because wealthier families might rely on alternative malaria prevention methods, which could reduce their utilization of insecticide-treated nets (ITNs).

The study found a positive correlation between ITN utilization and having a smaller family size (less than five). This may be due to a better balance between household size and the availability of ITNs, bedrooms, or separate sleeping areas. Studies from Ethiopia and Uganda have shown that using ITNs in single-room homes can be challenging due to limited space, making proper net hanging difficult [44, 47]. To enhance ITN use among larger families, it is recommended to ensure a consistent supply of ITNs, distribute them based on household size, and promote their proper utilization [37]. Discrepancies in ITN utilization across studies may be scientifically explained by differences in study design, population characteristics, ITN distribution and accessibility, household living conditions, and the effectiveness of health education interventions.

Pregnant women who attended at least one antenatal care (ANC) visit had significantly higher odds of ITN utilization compared to those who did not (AOR = 2.08; 95% CI: 1.21–2.58). This finding aligns with studies conducted in Uganda [38] and the Democratic Republic of Congo [48]. One possible explanation is that women who initially lacked an ITN received one from a health facility during an ANC visit after learning about its benefits. Additionally, ITN users were encouraged to use them, as doing so would benefit both themselves and their unborn children. Healthcare providers may have also warned them about the potential consequences of not using an ITN, reinforcing compliance [37]. Another possible reason for this result could be participation in interpersonal communication workshops held for pregnant women during antenatal care. Evidence suggested that providers’ counselling during ANC is very crucial to boost ITNs utilization [49, 50].

The findings of this study have clear policy and practical implications. They highlight the need for targeted ITN distribution strategies and context-specific health education, especially for pregnant women in rural areas. Health staff can use these insights to strengthen behavior change communication and integrate key messages into routine antenatal care and community outreach programs.

### Strengths and limitations of this study

The strengths of this study included its use of direct observation to confirm participants’ self-reported information regarding ITN utilization, check whether the nets were hung (mounted) over the sleeping places, and assess the physical condition of the nets. This study is limited by its cross-sectional design, which restricts the assessment of changes over time. Moreover, assessing ITN utilization based only on the previous night may not accurately represent long-term utilization patterns.

## Conclusion

The utilization of insecticide-treated nets (ITNs) among pregnant women in Dembecha District was 46.5%, which is significantly lower than the WHO’s recommended coverage level of 80% [51]. Antenatal care follow-up, having information about ITNs and their benefits, and educational status were significant factors influencing ITN utilization. Pregnant women who had routine ANC follow-up and those with a family size of fewer than five had a positive effect on increasing ITN utilization, while women with no education were less likely to use ITNs during pregnancy. To enhance ITN utilization and malaria prevention in Dembecha District, the government should strengthen antenatal care (ANC) services by integrating malaria education and ensuring consistent ITN distribution. Targeted interventions should include community-based training by health extension workers, regular household visits to monitor ITN usage, and culturally appropriate awareness campaigns delivered through local media, religious leaders, and health professionals.

## Data Availability

The datasets analysed during the current study are available from the corresponding author upon reasonable request.
